# The American Dental Association

**Published:** 1880-09

**Authors:** 


### EDITORIAL.

### THE AMERICAN DENTAL ASSOCIATION.

In the present number of the Register is presented a synopsis of the proceedings of the twentieth annual meeting of the American Dental Association. This was one of the most profitable and successful meetings of this body held for several years. The subjects were interesting, and profitably discussed. The attendance was larger than for several years; there were present one hundred and fifty-two members, (quite a number of ab-sentees sent dues) and quite a large number of visitors. The utmost harmony and good feeling prevailed throughout, and there seemed to be a warmer interest taken in the Association and its welfare than has often times been manifested. Then things were all encouraging and inspired hope for the future.

But it must be overlooked that these results came as the reward of judicious, well directed efforts. Dr. Shepard, at the meeting last year at Niagara, promised that an effort would be made for a good meeting at Boston. That promise was honored by Dr. S. and those who assisted him, and all shared the fruit. I cannot omit to mention the exceedingly pleasant entertainment provided for the members of the Association and their friends by the resident dentists, and by the Mayor and City of Boston. The former provided for and gave to the members a drive through Boston and its splendid suburbs. This occupied the afternoon of Wednesday, and though the weather was not propitious, the drive was a delightful one.

On Thursday afternoon the Mayor and City of Boston took the Association and other invited guests on a sail down the Harbor. A stop was made at Deer Island, and there was a variety of entertainment—beautiful concert singing by the children of the Reformatory Institute, and this followed by a most refreshing and acceptable collation at which Mayor Prince and one or two others extended most happy greetings.

These things added greatly to the social aspect of the meeting, and it is to be hoped that the examples set in the preparations for this annual gathering will not be overlooked in the future.

Of some special matters that were considered, something may be said in these pages hereafter.

